# Operative vs. nonoperative treatment in Mason type II fractures: a meta-analysis

**DOI:** 10.1016/j.xrrt.2026.100773

**Published:** 2026-05-07

**Authors:** Christian Goy, Bryan JM. van de Wall, Frank JP. Beeres, Björn C. Link, Niels M. van der Hoeven, Reto Babst, Yannic Lecoultre

**Affiliations:** a Faculty of Health Sciences and Medicine, University of Lucerne, Lucerne, Switzerland; b Department of Orthopedic and Trauma Surgery, Lucerne Cantonal Hospital, Lucerne, Switzerland; c Department of Health Sciences and Medicine, Leiden University, Leiden, The Netherlands

**Keywords:** Radial head fracture, Mason type II, ORIF, Operative, Nonoperative, Meta-analysis, Elbow fractures

## Abstract

**Background:**

It is unclear whether patients benefit from operative treatment in Mason type II radial head fractures. This meta-analysis aims to compare the 2 treatment modalities regarding functional outcomes, complications, and quality of life.

**Methods:**

A systematic search of PubMed, Embase, and the Cochrane Central Register of Controlled Trials was conducted for randomized controlled trials and observational studies comparing operative and nonoperative treatment for Mason type II fractures. Effect estimates were pooled using random-effects models. The primary outcome was elbow function, assessed by the Mayo Elbow Performance Score. Secondary outcomes included range of motion, complications, reinterventions, and quality of life.

**Results:**

Four studies, including 3 observational studies and 1 randomized controlled trial, with a total of 181 patients were included. No significant difference in the Mayo Elbow Performance Score was observed between the operative and nonoperative group (mean difference: 2.67; 95% confidence interval [CI]: −4.48-9.83; I^2^: 82%). Range of motion of elbow flexion, extension, pronation, and supination showed no significant difference. The nonoperative group showed no increased risk for overall complications at 9.2% vs. 23.4% (odds ratio: 2.66; 95% CI: 0.83-8.49; I^2^: 25%), or reinterventions at 5.7% vs. 11.7% (odds ratio: 1.29; 95% CI: 0.13-12.30; I^2^: 57%).

**Conclusion:**

This meta-analysis could not demonstrate a clear advantage of operative treatment for Mason type II fractures in terms of function or complications. However, the long-term effect of surgery in preventing osteoarthritis remains uncertain and should be considered in the treatment decision, as well as the degree of fracture displacement, even within the Mason type II spectrum. Further research focusing on long-term follow-up is needed to make a definitive statement in this regard.

Radial head fractures are considered one of the most common fractures of the elbow joint, accounting for approximately 30% of elbow fractures.^5^ Typically, younger patients are affected, with the mean age ranging from 38 to 49 years.^3^ A common classification for radial head fractures is the Mason classification.^4^^,^^10^ According to this system, radial head fractures are divided into 4 types. Type I fractures are undisplaced (<2 mm) and conservative treatment is warranted.^11^^,^^18^ Consensus is also found in Mason type III-IV fractures: patients with complete articular fractures (type III) or elbow dislocation (type IV) benefit from operative treatment. However, for Mason type II fractures (>2 mm displacement, partial articular fractures) (Fig. 1),^14^ there is no general agreement on the optimal treatment. Given the important role of the radial head as a stabilizer of the forearm and elbow, arguments for operative treatment include the potential benefit of an enhanced joint stability by operative restoration and potential prevention of osteoarthritis.^13^^,^^21^ Arguments against surgery include an expected higher incidence of complications due to surgical trauma and scar formation.^21^^,^^22^ For both nonoperative and operative treatment, good results have been reported.^1^^,^^6^, ^7^, ^8^ However, the individual studies lack the power to draw solid conclusions.

**Figure 1:**
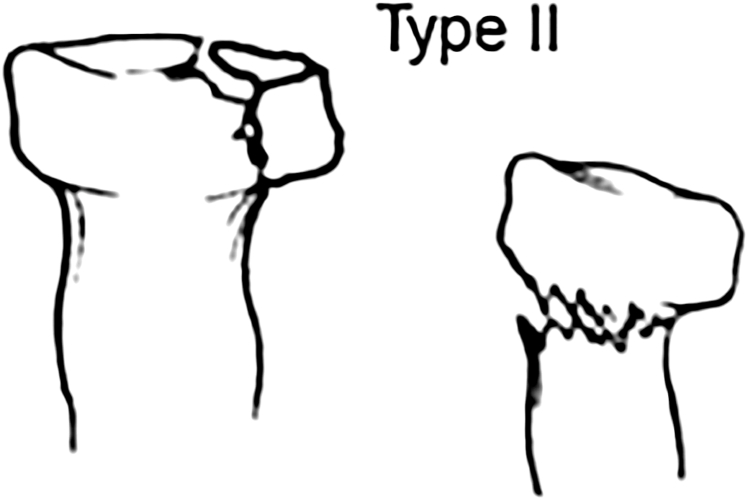
Illustration of Mason type II fractures.^14^

Hence, the aim of this study is to combine the outcomes of the individual studies into a meta-analysis with the goal of providing a treatment recommendation.

## Methods

This meta-analysis was conducted following the guidelines of the Preferred Reporting Items for Systematic Reviews and Meta-Analyses (Additional File 2) and the Meta-Analysis of Observational Studies in Epidemiology guidelines.^12^^,^^17^ A standardized methodology, consistently used in all meta-analyses by our research group, was employed.^9^^,^^20^ Ethical approval was not required. The study was recorded at the International Prospective Register of Systematic Reviews, Nr. CRD42024600904 prior to data collection.

### Search strategy and selection criteria

A comprehensive search was conducted in electronic databases (PubMed, Embase, and CENTRAL) for studies on treatment of Mason type II radial head fractures. The complete search syntax is detailed in Table S1 of the Additional File 1. The final search was carried out on September 1, 2024. All randomized controlled trials and observational studies that compared nonoperative to operative treatment of Mason type II radial head fractures were included. Additional inclusion criteria were reporting on the outcomes of interest as well as availability of full text. Exclusion criteria were cadaveric studies, studies on pathologic fractures, case reports, and studies with languages other than English, Dutch, French, German, Spanish, or Italian. Two reviewers (CG, YL) independently assessed the search results and study inclusions, with disagreements resolved through consensus with a third reviewer (BW).

### Data extraction

Study and patient characteristics were gathered using a predefined data extraction sheet. The collected information included the first author, journal title, publication year, the country or region where the study was conducted, as well as study design and study population size. Moreover, data on patient gender, age and, if available, history of smoking or diabetes were extracted.

### Quality assessment

The same 2 reviewers (CG, YL) independently evaluated the methodological quality of the included studies using the Methodological Index For Non-Randomized Studies criteria.^15^ Any disagreements were resolved through consensus. Further details are provided in Table S2 of the Additional File 1.

### Primary outcome

The primary outcome was long-term (>12 months) elbow function measured by the Mayo Elbow Performance Score (MEPS). The MEPS is a clinical assessment tool including pain, range of motion, stability, and daily function. The total score ranges from 0 to 100 points, with higher scores indicating better elbow function.

### Secondary outcomes

Secondary outcomes included elbow range of motion, complications, reinterventions, other functional elbow scores, and quality of life measured at 12 months or longer follow-up. Each component of range of motion was analyzed separately (flexion, extension, pronation, and supination). Complications encompassed heterotopic ossification, arthrofibrosis, early screw failure, intra-articular screw placement, as well as complex regional pain syndrome.^19^ Other functional elbow scores included the Oxford Elbow Score. Quality of life was measured with the Short Form 12 (SF-12) physical and mental.

### Statistical analysis

Continuous variables were reported as means with standard deviation (SD), range, or medians with interquartile range. Where necessary, data were transformed into mean and SD following the methods outlined in the Cochrane Handbook for Systematic Reviews of Interventions. Dichotomous variables were presented as counts and percentages. Formal meta-analysis was conducted only when 3 or more studies reported on the outcome of interest. The effects of different treatment options on continuous outcomes were pooled using the random-effects inverse variance weighting method and presented as mean difference (MD) with corresponding 95% confidence interval (95% CI). Binary outcomes were analyzed using the random-effects Mantel-Haenszel method and expressed as odds ratio (OR) with a 95% CI. Heterogeneity between studies was quantified using the I^2^ statistic. The threshold for significance was set at a *P* value of .05. Review Manager (RevMan, version 5.4.1) was utilized for statistical analysis.

## Results

### Literature search

A total of 466 references were evaluated. Detailed information on the search and screening process is provided in Table S1 of the Additional File 1. Three observational studies and 1 randomized controlled trial met the inclusion criteria.^7^^,^^11^^,^^21^^,^^22^

### Baseline characteristics

The 4 studies collectively included 181 patients, with 94 receiving operative treatment, of which 90 were treated with screw fixation. Only 4 patients received operative treatment with a T-plate. Baseline characteristics such as age, gender, mean follow-up, and study design are summarized in Table I. These characteristics were evenly distributed across treatment groups.

**Table I:** Baseline characteristics.

First author	Yr	Country	Design	Follow-up (mo)	Number of cases		Age mean (SD)		Gender (female, %)	
					OP	NOP	OP	NOP	OP	NOP
Khalfayan^7^	1992	USA	OS	18	10	16	38 (14.7)	41.4 (17.7)	50	25
Yoon^22^	2014	New Zealand	OS	45	30	30	39 (10)	51 (17)	46.7	70
von Glinski^21^	2019	Germany	OS	43	31	19	42.6 (13)	45.8 (11.5)	38.7	73.7
Mulders^11^	2021	Netherlands	RCT	12	23	22	50 (9.6)	50 (8.1)	60.9	50

*SD*, standard deviation; *NOP*, nonoperative; *OP*, operative; *OS*, observational study; *RCT*, randomized controlled trial.

### Quality assessment

The mean quality score of all studies, assessed using the Methodological Index For Non-Randomized Studies criteria, was 19.5 points (range, 18-22). Further details are available in Table S3 in the Additional File 1.

### Primary outcome

#### Mayo Elbow Performance Score

**Figure 2:**
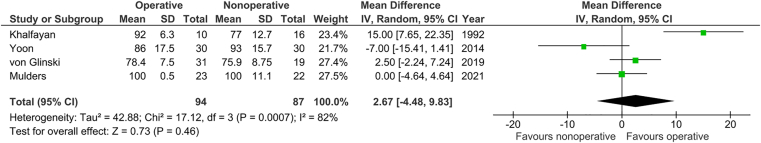
Forest plot of MEPS. *MEPS*, Mayo Elbow Performance Score.

The MEPS was reported in all 4 studies with a follow-up ranging from 12 to 45 months. No significant difference could be detected between the operative and nonoperative group (85.2 vs. 81.6 points; MD: 2.67; 95% CI: −4.48 to 9.83; I^2^: 82%) (Fig. 2).

### Secondary outcomes

#### Range of motion

**Figure 3:**
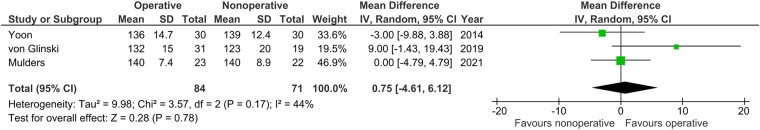
Forest plot of elbow flexion. *SD*, standard deviation; *CI*, confidence interval.

**Figure 4:**
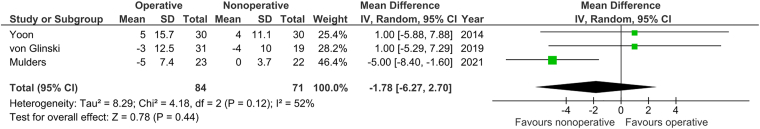
Forest plot of elbow extension. *SD*, standard deviation; *CI*, confidence interval.

**Figure 5:**
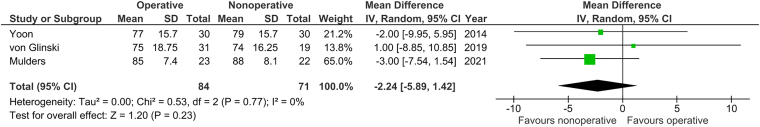
Forest plot of elbow supination. *SD*, standard deviation; *CI*, confidence interval.

**Figure 6:**
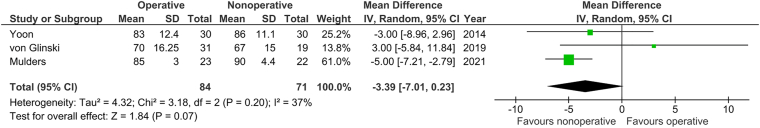
Forest plot of elbow pronation. *SD*, standard deviation; *CI*, confidence interval.

Three studies reported on the elbow range of motion, with no significant differences found between the operative and nonoperative group in terms of flexion (137.1° vs. 136.3°, MD: 0.75°, 95% CI: −4.61 to 6.12, I^2^: 44%), extension (−1.9° vs. −0.1°, MD: −1.78°, 95% CI: −6.27 to 2.70, I^2^: 52%), supination (82.0° vs. 84.2°, MD: −2.24°, 95% CI: −5.89 to 1.42, I^2^: 0%), and pronation (82.4° vs. 85.9°, MD: −3.39°, 95% CI: −7.01 to 0.23, I^2^: 37%) (Figure 3, Figure 4, Figure 5, Figure 6).

#### Complications

**Figure 7:**
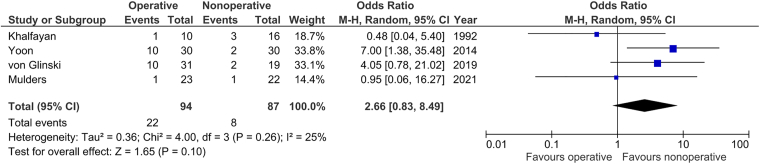
Forest plot of complications. *CI*, confidence interval; *M-H*, Mantel–Haenszel.

Complications were reported in all 4 studies. No significant difference could be found in overall complication rate between the operative and nonoperative group (23.4% vs 9.2%, OR: 2.66, 95% CI: 0.83-8.49, I^2^: 25%) (Fig. 7). The most common complications were heterotopic ossification (8.5% vs. 1.1%) and arthrofibrosis (7.4% vs. 0%), which mostly occurred in the operative group. Complications are described in Table II.

**Table II:** Complications.

First author	Yr	Number of cases		Heterotopic ossification		Arthro-fibrosis		Persistent pain		Early screw failure		Loose bodies		Arthrosis		Wound infection		CRPS	
		OP	NOP	OP	NOP	OP	NOP	OP	NOP	OP	NOP	OP	NOP	OP	NOP	OP	NOP	OP	NOP
Khalfayan^7^	1992	10	16	0	0	1	0	0	0	0	0	0	2	0	1	0	0	0	0
Yoon^22^	2014	30	30	8	1	0	0	0	0	2	0	0	0	0	0	0	0	0	1
von Glinski^21^	2019	31	19	0	0	6	0	2	0	0	0	0	0	0	0	0	0	0	0
Mulders^11^	2021	23	22	0	0	0	0	0	1	0	0	0	0	0	0	1	0	0	0
Total		**94**	**87**	**8**	**1**	**7**	**0**	**2**	**1**	**2**	**0**	**0**	**2**	**0**	**1**	**1**	**0**	**0**	**1**
Total (%)				8.5	1.1	7.4	0.0	2.1	1.1	2.1	0.0	0.0	2.3	0.0	1.1	1.1	0.0	0.0	1.1

*NOP*, nonoperative; *OP*, operative; *CRPS*, complex regional pain syndrome.

#### Reinterventions

**Figure 8:**
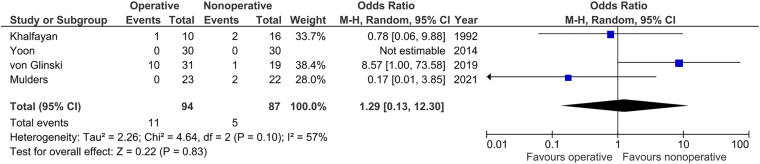
Forest plot of reinterventions. *CI*, confidence interval; *M-H*, Mantel–Haenszel.

All 4 studies reported on reinterventions. No significant difference could be detected between the operative and nonoperative groups, with 11 reinterventions in the operative group and 5 in the nonoperative group (11.7% vs. 5.7%, OR: 1.29, 95% CI: 0.13-12.30, I^2^: 57%) (Fig. 8). In the operatively treated group, the most common reason for reintervention was arthrofibrosis treatment (n = 7), while in the nonoperatively treated group, it was due to loose bodies in the elbow joint (n = 3), secondary dislocation (n = 1), and severe arthrosis (n = 1).

#### Other functional elbow scores

Two studies reported on the Oxford Elbow Score. Both studies showed no significant difference between the operative and nonoperative group. The mean scores for the operative group were 42.9 (SD: 4.3) and 48.0 (SD: 3.7), and for the nonoperative group 40.7 (SD: 5.0) and 46.0 (SD: 5.2).

#### Quality of life

One study reported on the SF-12. No significant differences could be shown. Mean scores for the SF-12 physical were 48 (95% CI: 45-50) for the operative and 51 (95% CI: 48-54) for the nonoperative group. The scores in the SF-12 mental respectively were 57 (95% CI: 54-59) and 54 (95% CI: 50-57).

## Discussion

This meta-analysis of 4 studies, including 3 observational studies and 1 randomized controlled trial, compared operative vs. nonoperative treatment of Mason type II fractures in adult patients. Operative fixation does not appear to significantly improve functional outcomes at 12-45 months follow-up. No difference in complication or reintervention rates and quality of life could be shown. However, when complications did occur, they were mostly related to surgery.

### Comparison to previous literature

To date, no meta-analysis has been conducted on this topic. A systematic review published in 2021 compared operative and nonoperative management of Mason type II fractures. The authors reported that both treatment modalities resulted in comparable functional outcomes, with no statistically significant differences. Ninety point nine percent of patients who underwent operative treatment achieved excellent or good outcomes, compared to 95.1% in the nonoperative group. However, the incidence of subsequent surgical interventions was higher among patients treated operatively (7.1% vs. 0%).^8^

### Interpretation of results

The results of this meta-analysis demonstrate no clear benefit for operative treatment in Mason type II fractures of the radial head. However, certain considerations should be taken into account.

No statistically significant or clinically relevant differences were found between the 2 treatment modalities in terms of functional scores at 12-45 months follow-up. While the follow-up duration is sufficient to draw conclusions about elbow function, it is not long enough to determine the occurrence of osteoarthritis. In a long-term follow-up study of Akesson et al,^1^ radiological osteoarthritic changes were found in 28 of 34 patients (82%) treated conservatively after a mean follow-up duration of 19 years. Approximately 77% of those patients, however, showed no clinical symptoms, making its clinical relevance questionable. Although long-term data are lacking, the risk of developing osteoarthritis in operatively treated patients with intra-articular fractures, such as radial head fractures, is directly related to the degree of fracture reduction achieved during initial surgery. It is plausible that, although surgery may show little measurable benefit in the first few years, its positive effect on preventing osteoarthritis could become apparent with longer follow-up.

It should also be pointed out that Mason type II fractures are solely defined by displacement greater than 2 mm irrespective of the actual amount of displacement measured on a continuous scale. It is well established that the amount of displacement correlates with the functional outcome and risk of developing osteoarthritis. A type II fracture with 3 mm displacement might not benefit as much from surgery as one does with 5 mm displacement. The results found in this study therefore apply to the general Mason type II population. In individual cases, however, the precise degree of displacement should still be carefully considered when making treatment decisions.

Even though not significant, complications such as heterotopic ossification and arthrofibrosis occurred almost exclusively in the operative group. This is consistent with the known negative effects caused by surgery. When looking at elbow range of motion, again there was a trend in favor of nonoperative treatment without reaching significance. These findings suggest that the damage caused by surgical intervention may potentially exceed that caused by a small joint incongruence treated conservatively.

The question that remains is whether the negative effect of surgery in the first months after surgery outweighs the potential beneficial effect on preventing osteoarthritis in the long-term. Until this is clarified, surgical treatment remains a viable option, especially as degree of dislocation increases within the Mason type II radial head fracture spectrum.

## Limitations

Several limitations must be considered in the interpretation of this study. Substantial heterogeneity of outcomes was observed between the studies. Also, the analysis included 3 observational studies and only 1 randomized controlled trial. While previous meta-analyses have shown that pooled estimates from observational studies approximate those from randomized controlled trials,^2^^,^^16^ we were unable to internally validate this assumption within the current meta-analysis. Furthermore, the total number of cases included was rather small, making it difficult to distinguish whether the observed lack of significance was due to insufficient statistical power or the actual absence of an effect. In addition, it is unclear to what extent fracture displacement in the observational studies included in this meta-analysis influenced treatment allocation and whether more displaced fractures were more frequently treated operatively. This introduces a potential risk of selection bias, which may have affected the comparability of the treatment groups.

## Conclusion

Based on the current data, no clear advantage of operative treatment for Mason type II fractures in terms of function could be demonstrated. The incidence of complications was low in both groups. However, when they did occur, they were mostly related to the surgical procedure itself. Since osteoarthritis may only become clinically apparent after long-term follow-up, the available evidence remains insufficient to determine whether operative fixation reduces the long-term risk of degenerative changes. Nevertheless, surgical restoration of joint congruity may reduce this risk, particularly in fractures with greater displacement. Given this uncertainty, it remains advisable to base the treatment decision on patient characteristics and the degree of fracture displacement, even within the Mason type II spectrum. Further research is needed to make definitive statements and better understand the long-term outcomes.

## Disclaimers:

Funding: No funding was disclosed by the authors.

Conflicts of interest: The authors, their immediate families, and any research foundations with which they are affiliated have not received any financial payments or other benefits from any commercial entity related to the subject of this article.
