# Supplementary material for: Operative vs. nonoperative treatment in Mason type II fractures: a meta-analysis
Source: JSES Rev Rep Tech. 2026 May 7;6(3):100773. doi: 10.1016/j.xrrt.2026.100773 (PMC13264368; doi:10.1016/j.xrrt.2026.100773)
Supplement: Additional File 1 [file mmc1.docx]

**Additional File 1**

**Operative vs. Nonoperative Treatment in Mason Type II Fractures: A Meta-Analysis**

Christian Goy^2*^, Bryan van de Wall^1,2^, Frank Beeres^1,2^, Bjoern-Christian Link^1^, Niels van der Hoeven^3^, Reto Babst^1,2^, Yannic Lecoultre^1,2^

1. Department of Orthopedic and Trauma Surgery, Lucerne Cantonal Hospital, Lucerne, Switzerland

2. Faculty of Health Sciences and Medicine, University of Lucerne, Lucerne, Switzerland

3. Department of Health Sciences and Medicine, Leiden University, Leiden, The Netherlands

* Corresponding author

E-mail: christian.goy@luks.ch

##

## S1 Table: Search Strategy / Syntax

| **Pubmed/MEDLINE (n=273)**  **Embase (n=156)**  **CENTRAL (n=37)**  **Total (n=466)** |  |
| --- | --- |
| **Title and abstract screening (n=13)** | Excluded by title / abstract: n=453 |
| **Full text articles assessed for eligibility (n=10)** |  |
| **Studies included (n=4)** |  |

| **Pubmed/MEDLINE** | ((((radius[Title/Abstract]) OR (radial[Title/Abstract])) AND ((head[Title/Abstract]) OR (neck[Title/Abstract]) OR (proximal[Title/Abstract]))) AND (fracture[Title/Abstract])) AND ((((((operati*[Title/Abstract]) OR (surgical[Title/Abstract])) OR (surgery[Title/Abstract])) OR (orif[Title/Abstract])) OR (open reduction[Title/Abstract])) AND (((nonoperativ*[Title/Abstract]) OR (non-surgical[Title/Abstract])) OR (conservativ*[Title/Abstract]))) |
| --- | --- |
| **Embase** | 'radius' AND ('head' OR 'neck' OR 'proximal radius fracture') AND ('surgery' OR 'open reduction (procedure)') AND 'conservative treatment' |
| **CENTRAL** | #1 fracture  #2 (radius OR radial) AND (head OR neck)  #3 surg*  #4 conservati* OR nonoperativ*  #5 #1 AND #2 AND #3 AND #4 |

## S2 Table: Quality assessment criteria

| **Criteria** | **Reported and adequate (2)** | **Reported but inadequate (1)** | **Not reported (0)** |
| --- | --- | --- | --- |
|  |  |  |  |
| Clearly stated aim | Aim including outcomes reported | Aim reported without outcomes | Not reported |
| Inclusion consecutive patients | Inclusion of consecutive patients | Clear description of inclusion criteria | Not reported |
| Prospective collection data | Prospective | retrospective | Not applicable |
| Appropriate endpoints | Appropriate endpoints to aim study | Endpoints not appropriate to aim study | Not reported |
| Unbiased assessment | Blinded evaluation of outcomes | Reason not blinding stated | Not reported |
| Appropriate follow-up | Minimum follow-up 12 months | Follow-up < 12 months | Not reported |
| Loss to follow-up < 5% | Less than 5 % | Reported, more than 5% | Not reported |
| Prospective calculation study size | Prospective power-analysis performed | Power analysis performed but inadequate | Not applicable |
| Adequate control group | Operative versus nonoperative treatment | Not applicable | Not applicable |
| Contemporary groups | Study/control group managed during same period | Study/control not managed during same period | Not reported |
| Baseline equivalence groups | Baseline characteristics described and comparable | Baseline characteristics not comparable | Not reported |
| Adequate statistical analyses | Statistical analysis described including type of analyses | Inadequate description statistical analysis | Not reported |

## S3 Table: Quality assessment

|  | Khalfayan^7^ | Yoon^22^ | Von Glinski^21^ | Mulders^11^ |
| --- | --- | --- | --- | --- |
| **Clearly stated aim** | 2 | 2 | 2 | 2 |
| **Inclusion of consequetive patients** | 2 | 2 | 2 | 2 |
| **Prospective data collection** | 1 | 1 | 1 | 2 |
| **Appropriate endpoints** | 2 | 2 | 2 | 2 |
| **Unbiased assessment endpoints** | 0 | 0 | 0 | 1 |
| **Appropriate follow-up** | 2 | 2 | 2 | 2 |
| **Loss-to-follow-up <5%** | 1 | 1 | 1 | 1 |
| **Prospective calculation study size** | 0 | 2 | 0 | 2 |
| **Adequate control group** | 2 | 2 | 2 | 2 |
| **Contemporary groups** | 2 | 2 | 2 | 2 |
| **Baseline equivalence of groups** | 2 | 2 | 2 | 2 |
| **Adequate statistical analysis** | 2 | 2 | 2 | 2 |
| **Total:** | 18 | 20 | 18 | 22 |
